# Electrochemical and Mechanical Performance of Magnetron-Sputtered AlCrFeVTi High-Entropy Alloy Coatings for Lead-Cooled Fast Reactors

**DOI:** 10.3390/ma19051006

**Published:** 2026-03-05

**Authors:** Shahid Ali, Zahid Hussain, Abdalelah H. Balal, Yuefei Jia, Naeem ul Haq Tariq, Aiman Mukhtar, Gang Wang

**Affiliations:** 1 State Key Laboratory of Materials for Advanced Nuclear Energy, Shanghai University, Shanghai 200444, China; shahidali@shu.edu.cn; 2 Zhejiang Institute of Advanced Materials, Shanghai University, Jiashan 314100, China; 3 Department of Mechanical Engineering, University of Engineering and Technology Lahore, Narowal Campus, Narowal 51600, Pakistan; zahid.hussain@uet.edu.pk; 4 Institute of Materials, Shanghai University, Shanghai 200444, China; 5 Department of Metallurgy and Materials Engineering, Pakistan Institute of Engineering and Applied Science (PIEAS) Nilore, Islamabad 45650, Pakistan; naeem421@hotmail.com; 6 The State Key Laboratory of Refractories and Metallurgy, Hubei Province Key Laboratory of Systems Science in Metallurgical Process, International Research Institute for Steel Technology, Wuhan University of Science and Technology, Wuhan 430081, China

**Keywords:** high-entropy alloy, amorphous coating, corrosion resistance, EIS, nanoindentation, mechanical properties, coating, nuclear applications

## Abstract

High-entropy amorphous materials are attracting increasing attention due to their excellent corrosion resistance and radiation tolerance in nuclear environments. In this study, novel Al_2_Cr_16_Fe_50_V_20_Ti_12_ high-entropy alloy (HEA) coatings with thicknesses of 900 nm and 1400 nm were synthesized via magnetron sputtering and systematically evaluated for their structural, electrochemical, and mechanical performance. X-ray diffraction confirmed the amorphous nature of the coatings, while scanning electron microscopy revealed a denser, defect-free, and more uniform morphology in the thicker coating. Electrochemical testing in a 3.5 wt.% NaCl solution demonstrated a tenfold reduction in corrosion current density and nearly a twofold increase in charge transfer resistance for the 1400 nm coating, attributed to its improved passive film stability. Finite element modeling validated the experimental load–displacement behavior and revealed well-confined and uniformly distributed stress and strain fields within the coating. These findings establish the 1400 nm Al_2_Cr_16_Fe_50_V_20_Ti_12_ coating as a promising candidate for protective applications in chloride-rich and radiation-intense nuclear systems.

## 1. Introduction

Several nations established the Generation IV International Forum (GIF) to promote the development of advanced nuclear systems to meet future global energy demands. Its goal is to facilitate the establishment of fourth-generation nuclear power technologies by 2030, coinciding with the retirement of many existing nuclear reactors. Among the six most promising Gen-IV designs—GFR (gas-cooled fast reactor), LFR (lead-cooled fast reactor), MSR (molten salt reactor), SFR (sodium-cooled fast reactor), SCWR (supercritical water-cooled reactor), and VHTR (very-high-temperature reactor)—the lead-cooled fast reactor (LFR) offers strong advantages in sustainability, safety, and economics due to its use of a closed fuel cycle and long-life core [1]. The use of lead-based coolants with low melting and high boiling points enhances safety by reducing the risk of an LOCA (loss-of-coolant accident) and improving thermal efficiency [2]. Liquid lead–bismuth eutectic (LBE) is a particularly attractive coolant candidate for LFRs due to its favorable thermophysical and neutron properties [3]. However, its corrosive nature poses serious threats to structural materials, especially at high temperatures. Austenitic stainless steels and ferritic/martensitic (F/M) steels exposed to LBE are prone to local and uniform degradation, affecting their long-term reliability [4,5,6,7]. Surface coatings have emerged as a promising solution to enhance corrosion resistance without altering the base microstructure. These include ceramic coatings (Al_2_O_3_ [8,9], Y_2_O_3_, SiC, AlTiN, TiSiN) or metallic coatings (FeAl, FeCrAl, FeAlTi, FeCrAlY) [10]; both materials have been thoroughly studied and shown to offer good resistance to LBE corrosion. However, ceramic coatings face challenges such as weak adhesion, low thermal conductivity, and difficulties in processing, which restrict their use in protecting against LBE corrosion [11,12,13]. Metallic coatings can effectively address the issues mentioned earlier. HEA coatings, a new type of metallic coating [14], demonstrate enhanced overall performance compared to conventional metallic coatings due to their unique multi-principal element structures and the “four effects” (high entropy effect, lattice distortion effect, cocktail effect, and sluggish diffusion effect) [15,16,17,18]. Nevertheless, studies on HEA coatings for LBE corrosion protection are still limited [10]. The current HEA coatings primarily consist of various protective elements (such as Al, Cr, and Ti), which form a protective oxide layer on the surface to prevent oxidative corrosion during LBE exposure [19,20,21].

In oxygen-saturated LBE environments, corrosion is dominated by oxidation reactions forming protective oxide layers, while in oxygen-depleted LBE, dissolution of metal elements becomes the primary degradation mechanism [22]. Although coatings like AlCrFeVTi HEA have shown enhanced resistance in LBE, the role of oxygen concentration in optimizing passivation remains unclear [3]. The corrosion mechanisms in NaCl versus LBE environments differ significantly. In chloride-rich NaCl solutions, degradation occurs primarily through localized breakdown of the passive film, leading to pitting corrosion. Chloride ions aggressively attack passive films, initiating pitting at defects and heterogeneities, which makes NaCl testing particularly effective for evaluating coating uniformity and defect density. In contrast, LBE corrosion is governed by fundamentally different mechanisms. At high oxygen concentrations, corrosion proceeds through oxidation of the steel, forming protective oxide layers [23]. At low oxygen concentrations, elemental dissolution becomes the primary degradation mechanism, where alloying elements selectively dissolve into the liquid metal [22]. Therefore, while excellent performance in NaCl indicates a dense, high-quality coating with stable passivation, a necessary prerequisite for LBE resistance, it does not guarantee equivalent performance in LBE without direct validation. As noted by Martinelli et al. [23], the oxidation-controlled regime in LBE requires specific testing conditions that account for oxygen concentration, temperature, and flow dynamics, which we identify as a critical future research direction. They offer exceptional resistance to corrosion [24], irradiation, and thermal degradation while maintaining mechanical robustness [25,26]. Their cost-effective fabrication has increased their appeal for nuclear applications [10]. HEAs are seen as materials with great potential for various demanding applications in the nuclear industry [10]. For instance, Zhang et al. developed a CoCrCuFeNi HEA coating and discovered that the phase structure of the coating remained highly stable up to 750 °C. Additionally, the coating retained high hardness even after annealing at 1000 °C for 5 h [27]. Takeshi et al. reported that the CoCrCuFeNi HEA coating showed excellent structural stability under electron irradiation across a broad temperature range from 298 to 773 K. The main constituent phase remained intact even when the samples were irradiated up to 40 displacements per atom (dpa) [28]. Cui et al. deposited an AlCoCrFeNiTi HEA coating, which demonstrated high hardness and excellent high-temperature oxidation resistance due to the formation of continuous Cr2O3/Al2O3 oxide scales [29]. Li et al. successfully fabricated an FeAlCuCrCoMn HEA coating exhibiting remarkable corrosion resistance in diverse corrosive environments [30]. Such exceptional corrosion-resistant properties position HEA coatings as strong candidates for enhancing the surface durability of structural components in LBE-cooled fast reactors.

Various methods have been used to deposit HEA coatings [31], including thermal spray [32], laser cladding [33] and magnetron sputtering [34]. Magnetron sputtering is especially suitable for nuclear coatings due to its ability to deposit films at room temperature, preserving the base material. It also enables precise control of thickness and composition, making it feasible for full-scale fuel cladding applications [3]. In this study, an Al_0.02_Cr_0.16_Fe_0.5_V_0.2_Ti_0.12_ high-entropy alloy was investigated, chosen for its optimized combination of corrosion resistance, mechanical strength, and irradiation tolerance. Each element is selected for its unique function: Cr and V enhance oxidation and structural stability, Fe contributes strength, Al enables passive coating formation while reducing density, and Ti improves creep and fatigue resistance—key attributes for nuclear applications. The amorphous HEA coatings were deposited using direct current (DC) magnetron sputtering, employing a mosaic target composed of five high-purity elemental segments. This approach enabled fine-tuned control of elemental composition through independent adjustment of magnetron power for each source. The coating was grown on continuously rotating Si (100) substrates under high-vacuum conditions to ensure uniformity and control over thickness. Two specific novel HEA alloy coating thicknesses, 900 nm and 1400 nm (achieved by varying deposition time), were selected to systematically investigate how deposition duration and material accumulation affect the microstructure, corrosion performance, and mechanical properties of the coatings.

By integrating advanced electrochemical characterization, depth-sensitive nanoindentation analysis, and finite element modeling, this work aims to elucidate the structure–property relationships governing the performance of a novel HEA coating in aggressive chloride-containing environments. The findings will offer critical insights for designing next-generation protective coatings capable of withstanding the harsh thermomechanical and chemical challenges in lead–bismuth eutectic-cooled nuclear systems. It should be noted that, while LBE corrosion involves complex oxidation and dissolution mechanisms, the present study employs a standardized 3.5 wt% NaCl electrolyte. This approach enables a fundamental, comparative assessment of the intrinsic electrochemical barrier properties of coatings and passive film stability in a well-controlled aggressive environment. The use of NaCl as a testing electrolyte follows established methodology for evaluating protective coatings for nuclear applications, where standardized chloride testing enables quantitative comparison of coating quality and passive film stability before proceeding to application-specific environments [35].

This study presents the first comprehensive investigation of novel Al_2_Cr_16_Fe_50_V_20_Ti_12_ high-entropy alloy coatings with thicknesses of 900 nm and 1400 nm, deposited via magnetron sputtering. The novelty of this work lies in three key aspects: (i) the successful synthesis of a previously unreported non-equiatomic amorphous HEA composition specifically tailored for nuclear applications; (ii) the integrated methodology coupling electrochemical characterization with experimentally validated finite element nanoindentation modeling, enabling unprecedented insight into both corrosion mechanisms and subsurface mechanical behavior; and (iii) the systematic thickness-dependent analysis establishing clear relationships between deposition parameters, microstructure, and performance.

## 2. Materials and Methods

### 2.1. Materials

A high-purity (99.95%) composite target of Al_2_Cr_16_Fe_50_V_20_Ti_12_ was purchased from Deyang ONA New Materials Co., Ltd. (Deyang, China), and used as the reference material for the magnetron sputtering process. Monocrystalline silicon wafers (5 × 5 × 1 mm^3^), obtained from Kaihua Lijing Electronics Co., Ltd. (Quzhou, China), were used as substrates for coating deposition. The fitting procedure employed a complex nonlinear least squares method in ZsimpWin software (version 4.0, AMETEK Scientific Instruments, Berwyn, PA, USA), with chi-squared values < 0.001 indicating excellent fit quality. Each EIS test was conducted in triplicate, and the extracted parameters remained within ±0.5% standard deviation, confirming reproducibility.

### 2.2. Coating Deposition

Al_2_Cr_16_Fe_50_V_20_Ti_12_ coatings with thicknesses of ~900 nm and ~1400 nm were deposited using a DC magnetron sputtering system (JPG-450, Shenyang ZKY Technology Development Co., Ltd., Shenyang, China). Deposition was carried out under a base vacuum of 5 × 10^−5^ Pa. High-purity argon gas (99.999%) was introduced at a flow rate of 80 sccm, maintaining a working pressure of 0.3 Pa. The sputtering power was fixed at 50 W. Prior to deposition, a 30 min pre-sputtering cycle was applied to remove surface contaminants from the target. The target–substrate distance was set at 80 mm. Deposition times of 2 h and 3 h produced coatings of ~900 nm and ~1400 nm, respectively. These parameters were optimized through preliminary trials to achieve uniform coatings with controlled thickness. A summary of the sputtering conditions is provided in Table 1.

### 2.3. Microstructural Characterization

The morphological properties, including surface and cross-sectional properties, of the Al_2_Cr_16_Fe_50_V_20_Ti_12_ coatings were examined using a field emission scanning electron microscope (FESEM, Zeiss Gemini 300, Carl Zeiss AG, Oberkochen, Germany) set in the secondary electron mode, 5 kV accelerating voltage, and 5 mm working distance. The coating specimens were glued onto flat sample holders for study. The coating, before measurement, was cleaned thoroughly with ethanol in order to eliminate the presence of surface contaminants that could interfere with the outcome. A protective coating was not used since the coating could interfere with the diffraction properties. The X-ray diffraction (XRD) patterns of the coating were recorded in the open air of the laboratory at room temperature using Cu-Kα radiation (λ = 1.5406 Å) in the 2θ range of 20–80°. In order to reduce the effect of the rough surface of the sample on the resultant diffraction output, the sample was rotated when the scanning was done. The obtained XRD data were normalized in terms of intensity variation to enable accurate identification of phases. The phases of the coating were identified using an X-ray diffractometer (XRD, Rigaku D/MAX-2500, Tokyo, Japan) in conjunction with Cu-Kα radiation (λ = 1.5406 Å) with 30-kV operative voltage. The scan of the 20–80° range was done in 5°/min speed. The surface morphology of the high-entropy alloys (HEAs) coating was studied using a setup of field emission transmission electron microscopy (TEM, JEM-F200, JEOL Ltd., Tokyo, Japan) set in 200 kV. Selected area electron diffraction (SAED) methods were used to analyze the phase stability. The TEM samples, in the form of cross-sectional samples, were made through the use of focused ion beam milling (FIB, FEI, Quanta 3D FEG, FEI Company, Brno, Czech Republic) through the use of Ga^+^ ion source set in 30 kV. The elemental content and spatial distributions of the products were identified using energy-dispersive X-ray spectroscopy (EDS) coupled with JEOL JSM-7500F field emission scanning electron microscopy.

### 2.4. Electrochemical Testing for Corrosion Resistance

#### 2.4.1. Potentiodynamic Polarization Test

To evaluate the corrosion resistance of the high-entropy alloy (HEA) coating, potentiodynamic polarization tests were carried out in a 3.5 wt% sodium chloride (NaCl) solution at ambient temperature. A conventional three-electrode setup was utilized, where the HEA-coated sample (exposed area: 5.30 mm^2^) served as the working electrode, a platinum plate was used as the counter electrode, and a saturated calomel electrode (SCE) acted as the reference. Prior to initiating the polarization measurements, the open circuit potential (OCP) was recorded for 120 min to allow the system to stabilize. The polarization curve was obtained by scanning the potential from −1.0 V to +1.0 V relative to SCE at a rate of 1 mV/s [36]. The corrosion current density (I_corr_) and corrosion potential (E_corr_) were extracted through Tafel plot extrapolation. The corrosion rate was then calculated in accordance with the ASTM G102 standard [37] using the following equation (Equation (1)):

(1) $$CR=K\times \left(\frac{I_{corr}}{\rho }\right)\times (EW)$$

In this equation, K represents a constant with a value of 3.27 × 10^−3^ mm·g·µA^−1^·cm^−1^·year^−1^, EW denotes the equivalent weight of the HEA alloy, and ρ refers to the material’s density, which was estimated based on the rule of mixtures. To ensure accuracy and reproducibility, each Tafel test was repeated 3 times, and the extracted corrosion parameters remained within a standard deviation of ±1%.

#### 2.4.2. Electrochemical Impedance Spectroscopy (EIS)

Electrochemical impedance spectroscopy (EIS) tests were conducted using the same three-electrode configuration detailed in Section 2.4.1. The impedance spectra were acquired across a frequency ranging from 10^2^ kHz to 10^−5^ kHz using an alternating voltage signal with an amplitude of 10 mV [36]. Nyquist and Bode plots were generated to interpret the electrochemical behavior of the coating, and equivalent electrical circuit (EEC) models comprising resistive and constant phase elements (CPEs) were used to fit the impedance spectra using Z simp View (version 4.0, AMETEK Scientific Instruments, Berwyn, PA, USA). Important parameters including charge transfer resistance (R_ct_) and coating resistance (R_s_) were extracted, with higher R_ct_ values indicating improved corrosion resistance [38]. Each EIS test was conducted in triplicate to ensure data reliability and repeatability.

### 2.5. Mechanical Properties Investigation: Nanoindentation Testing

Nanoindentation tests were carried out using a KLA iMicro nanoindenter (KLA Corporation, Milpitas, CA, USA) with a Berkovich diamond tip. To minimize substrate effects, indentation depths were limited to 140 nm for 1400 nm coating, respectively. A constant strain rate of 0.05 s^−1^ was applied, and each indent was held at peak load for 5 s. Five indents per sample ensured statistical reliability. Hardness and reduced modulus were calculated using the Oliver–Pharr method [39].

**Figure 1:**
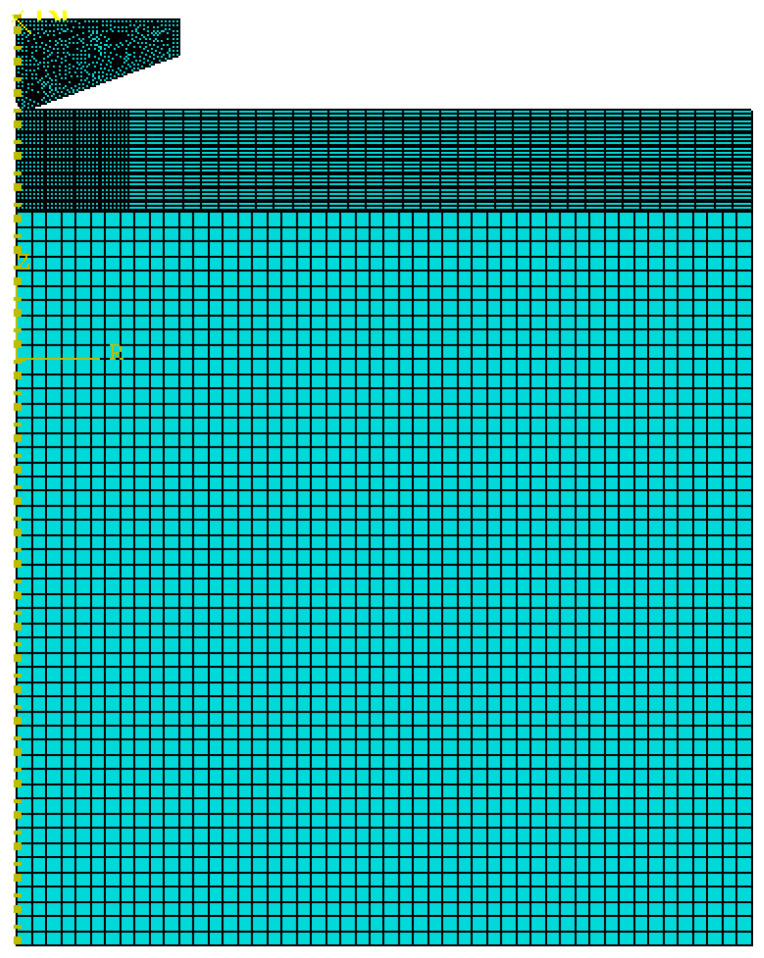
Finite element mesh of the 2D axisymmetric model showing the indenter, Al_2_Cr_16_Fe_50_V_20_Ti_12_ HEA coating, and sapphire substrate.

To complement the experimental results and provide insight into subsurface stress–strain evolution, finite element analysis (FEA) of the nanoindentation process was performed using ABAQUS/CAE 2021. A two-dimensional axisymmetric model was constructed to represent the Berkovich indenter modeled as a rigid conical body with a semi-apex angle of 70.3° along with the Al_2_Cr_16_Fe_50_V_20_Ti_12_ HEA coating and sapphire substrate, as shown in Figure 1. This modeling approach enabled efficient simulation of the contact mechanics while capturing the essential deformation behavior.

The coating and substrate were assigned elastic–plastic material properties based on experimental nanoindentation data and supplemented with literature values [40] (see Table 2). A frictionless surface-to-surface contact interaction was defined between the indenter and the coating surface, and a tie constraint was applied at the coating–substrate interface to ensure full adhesion. Displacement-controlled loading was applied to the indenter, and load–displacement (P–h) curves were recorded by tracking the indenter’s reaction force at incremental indentation depths.

The finite element mesh consisted of approximately 5000 quadrilateral axisymmetric reduced-integration elements (CAX4R) (Figure 1). The coating was discretized using ~1920 structured elements, while the substrate was modeled with ~2500 elements, coarsened progressively with distance from the contact interface to reduce computational expense. A high-resolution mesh was applied in the contact region, with element sizes refined to 2–5 nm near the coating surface to accurately capture steep gradients in stress and plastic strain. A mesh sensitivity analysis was conducted by varying element sizes and comparing the resulting P–h curves and von Mises stress profiles. The selected mesh resolution yielded less than 2% deviation from the finest mesh case, indicating convergence and mesh independence of the numerical results. Model accuracy was further verified by comparing the simulated P–h curves with experimental nanoindentation data, showing excellent agreement in both maximum depth and unloading stiffness [41]. Once validated, the model was employed to estimate yield strength by iterative fitting and to extract von Mises stress and equivalent plastic strain distributions, which helped identify subsurface deformation mechanisms.

**Table 2:** Physical and mechanical properties of indenter and substrate.

Module	Substance	Characteristics
Indenter	Diamond [42]	Elastic Modulus: 1,140,000 MPa Poisson’s ratio: 0.07
Substrate	Sapphire [42]	Elastic Modulus: 450,000 MPa Poisson’s ratio: 0.27 Initial yield stress: 8700 MPa

Although nanoindentation experiments were performed on coatings deposited on silicon wafers, sapphire was used as an ideal elastic substrate in the FEM simulations. Since the maximum indentation depth (≤140 nm) was limited to approximately 10% of the coating thickness, deformation was primarily confined within the coating, thereby minimizing substrate influence. The excellent agreement between simulated and experimental load–displacement curves confirms that this simplification does not affect the extraction of intrinsic coating properties. Similar modeling approaches have been reported in the literature [39,43,44].

## 3. Results and Discussions

### 3.1. Structural Analysis of Al_2_Cr_16_Fe_50_V_20_Ti_12_ Coating

**Figure 2:**
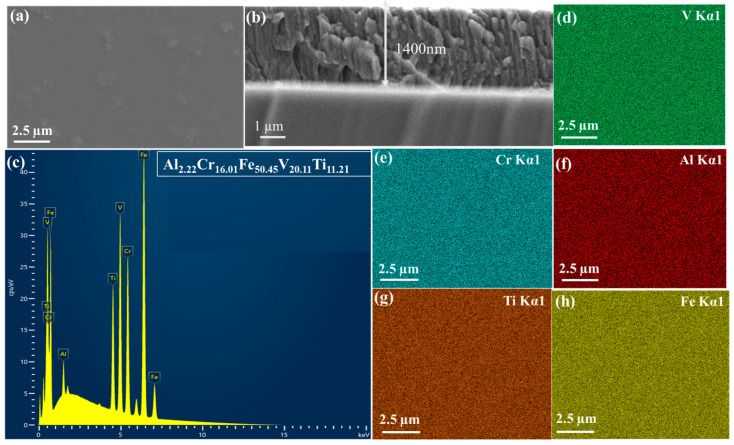
Typical cross-section image and chemical composition of the as-deposited HEA Al_2_Cr_16_Fe_50_V_20_Ti_12_ coating; (**a**) selected surface for EDS characterization; (**b**) cross-sectional view of characterized HEA coating; (**c**) obtained EDS spectrum; (**d**–**h**) elemental map of Al_2_Cr_16_Fe_50_V_20_Ti_12_.

**Figure 3:**
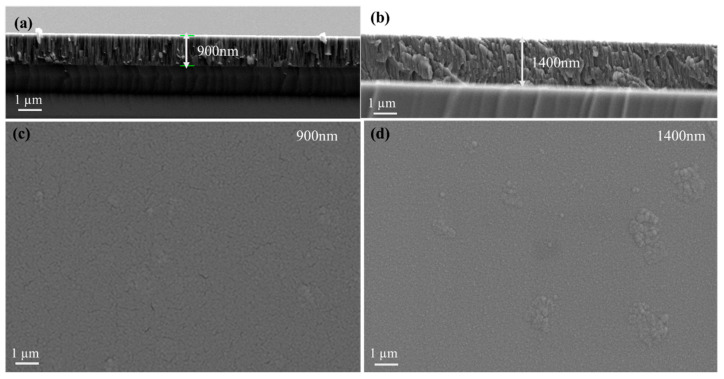
Cross-sectional and surface SEM images of Al_2_Cr_16_Fe_50_V_20_Ti_12_ HEA coating: (**a**) cross-sectional view of 900 nm coating showing visible columnar growth and microstructural irregularities; (**b**) cross-sectional view of 1400 nm coating exhibiting a denser, more uniform microstructure with increased thickness and improved deposition quality; (**c**) surface morphology of 900 nm coating revealing relatively rough surface with nodular features and incomplete coalescence; (**d**) surface morphology of 1400 nm coating displaying smooth, compact surface with uniform morphology and minimal defects.

The EDS (Figure 2) surface and cross-sectional SEM analysis provided critical insights into the microstructural characteristics of the Al_2_Cr_16_Fe_50_V_20_Ti_12_ HEA coating. Figure 3 presents the cross-sectional morphology of the as-deposited coatings, suggesting thicknesses of approximately 900 nm and 1400 nm, respectively. Elemental composition, measured via energy-dispersive X-ray spectroscopy (EDS), is summarized in Table 3.

Both coatings exhibit compositions closely matching the target stoichiometry, with minor deviations likely arising from kinetic factors during deposition, such as element-specific sputtering yields or re-sputtering effects [10]. These results confirm the successful fabrication of a near-equiatomic Al_2_Cr_16_Fe_50_V_20_Ti_12_ coating using the magnetron sputtering process. As shown in Figure 3, the 1400 nm coating exhibits a dense, continuous, and uniform microstructure with minimal surface defects, indicating improved nucleation and growth stability during prolonged deposition. In contrast, Figure 3c reveals that the 900 nm coating has relatively porous and discontinuous morphology, with visible surface irregularities and micro-defects. This difference in surface quality is likely due to insufficient deposition time and adatom mobility in the thinner coating, which may hinder complete surface coverage and grain coalescence. The longer sputtering duration for the 1400 nm coating promotes enhanced atom diffusion and layer densification, contributing to a more uniform and defect-free structure. At the initial stages of physical vapor deposition, coating growth often proceeds via the Volmer–Weber mechanism, where atoms preferentially bond to themselves rather than the substrate, forming isolated 3D islands. Over time, with increasing deposition, these islands coalesce into a continuous coating. The 900 nm coating likely reflects an incomplete coalescence stage, while the 1400 nm coating represents a more advanced, post-coalescence regime with uniform coverage.

**Figure 4:**
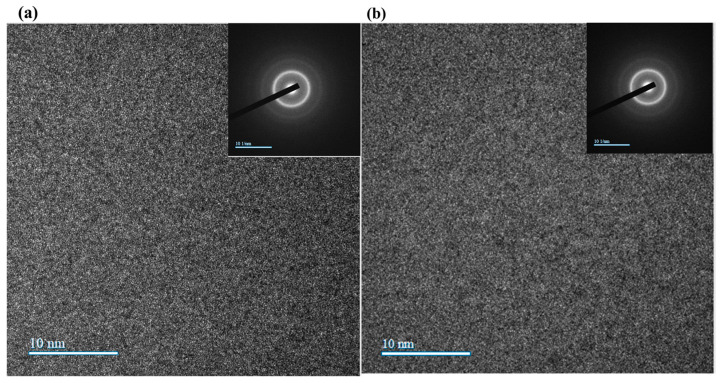
TEM analysis of Al_2_Cr_16_Fe_50_V_20_Ti_12_ HEA coating: (**a**) 900 nm coating showing interface waviness and contrast inhomogeneity; (**b**) 1400 nm coating displaying uniform contrast and dense amorphous structure. SAED patterns in both coatings confirm fully amorphous character.

The corresponding HRTEM observations and SAED patterns are shown in Figure 4a,b. Both coatings maintain a fully amorphous structure, as confirmed by the broad, diffuse halos in the SAED patterns. However, clear structural differences are observed between the two coatings. Small contrast variations and waviness are observed throughout the cross-section of the 900 nm coating, especially near the coating–substrate interface. These features indicate density fluctuations and potential nanoscale porosity in the 900 nm coating, indicating its incomplete or irregular growth during the sputtering process. In contrast, the 1400 nm coating in Figure 4b displays a smooth and homogeneous amorphous contrast (having a diffused SAED pattern), with no apparent density fluctuations or morphological defects. These differences reinforce the role of increased thickness in promoting a more stable microstructure, supporting superior electrochemical performance and improved environmental durability.

### 3.2. Corrosion Resistance of Al_2_Cr_16_Fe_50_V_20_Ti_12_ Coating

**Figure 5:**
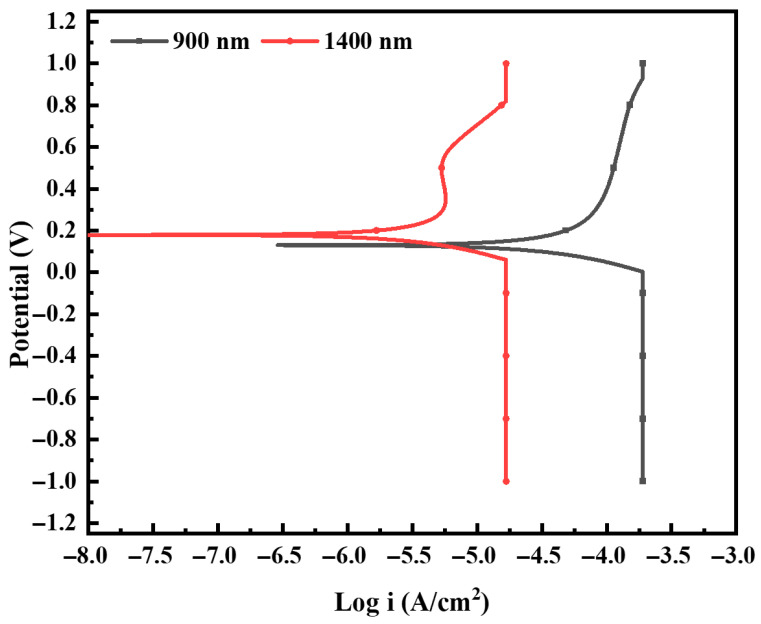
Potentiodynamic polarization curves of Al_2_Cr_16_Fe_50_V_20_Ti_12_ HEA coatings in 3.5 wt% NaCl solution.

The potentiodynamic polarization behavior of the Al_2_Cr_16_Fe_50_V_20_Ti_12_ HEA coating is illustrated in Figure 5, which presents the potential (V) versus logarithmic current density (Acm^−2^) on a semi-logarithmic Tafel plot. The polarization curve of the 1400 nm coating exhibits a distinct positive shift in corrosion potential (E_corr_) and a significantly lower corrosion current density (I_corr_) compared to the 900 nm coating, indicating its superior electrochemical stability in a 3.5 wt% NaCl environment. Quantitatively, the I_corr_ of the 1400 nm coating is 4 µAcm^−2^, while that of the 900 nm coating is substantially higher at 44.6 µAcm^−2^. This nearly tenfold reduction in current density demonstrates that the thicker coating offers significantly better resistance to electrochemical degradation. The plateau observed in the cathodic branch is attributed to diffusion-limited oxygen reduction, resulting in a limiting current density in the aerated NaCl solution. Corrosion rates (CR), calculated in accordance with ASTM G102 using Equation (1), were 0.5732 mm·year^−1^ for the 900 nm coating and 0.0514 mm·year^−1^ for the 1400 nm coating. These values correlate directly with the I_corr_ results, further confirming the markedly improved corrosion resistance of the thicker HEA coating. Additionally, the 1400 nm coating exhibits a broader passive region, evident as a plateau in the anodic branch from approximately +0.1 V to +0.6 V, suggesting the formation of a stable and protective oxide layer. This passive behavior is either absent or significantly less pronounced in the 900 nm coating, which shows a steep increase in current density, indicative of active corrosion and possibly localized breakdown. The enhanced performance of the 1400 nm coating is attributed to its dense and uniform microstructure (Figure 3), which acts as a robust barrier against electrolyte ingress and reduces the number of defect-induced active corrosion sites. In contrast, the 900 nm coating contains morphological defects such as pinholes and discontinuities that facilitate the initiation and propagation of localized corrosion. Overall, the results demonstrate that increasing the coating thickness not only reduces corrosion current and rate but also promotes passivation, making the 1400 nm Al_2_Cr_16_Fe_50_V_20_Ti_12_ coating a more suitable candidate for applications in aggressive corrosive environments.

**Figure 6:**
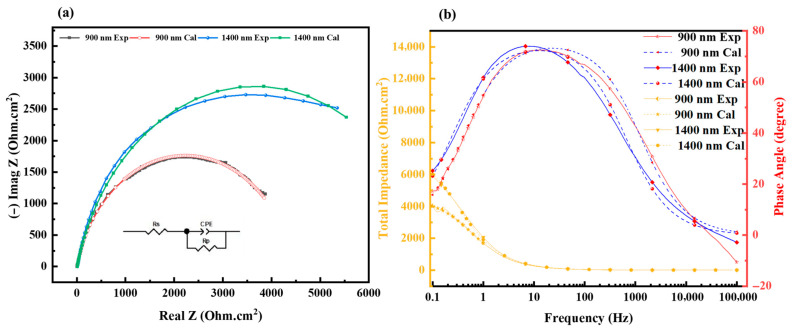
EIS results of Al_2_Cr_16_Fe_50_V_20_Ti_12_ HEA coatings in 3.5 wt% NaCl: (**a**) Nyquist plot with EEC model; (**b**) Bode plots.

Figure 6 presents the electrochemical EIS results for the Al_2_Cr_16_Fe_50_V_20_Ti_12_ coatings of 900 nm and 1400 nm thicknesses. The Nyquist plots (Figure 6a) show that the 1400 nm coating exhibits a significantly larger semicircle diameter compared to the 900 nm coating, indicating a higher charge transfer resistance (R_ct_) and, hence, improved corrosion resistance. The equivalent electrical circuit (EEC) used to fit the EIS data is shown in the inset of Figure 6a. It comprises the solution resistance (Rs), a constant phase element (CPE), and a polarization resistance (Rp) representing the charge transfer resistance. The close agreement between experimental and fitted data in both Nyquist and Bode plots confirms the suitability of the selected model, with a chi-squared value close to 1, indicating an excellent fit. The reported Rct values of 4.369 Ω·cm^2^ and 8.429 Ω·cm^2^ for the 900 nm and 1400 nm coatings, respectively, represent area-normalized charge transfer resistances (raw resistance values of 82.4 Ω and 159.0 Ω multiplied by the exposed surface area of 0.053 cm^2^). The relatively low magnitude of these values is attributed to two factors: first, the high conductivity of the 3.5 wt% NaCl electrolyte, which minimizes solution resistance; and second, the metallic nature of the coating surface, which facilitates charge transfer compared to insulating oxide coatings. Critically, while the absolute values are modest, the nearly twofold increase for the 1400 nm coating (8.4 Ω·cm^2^ vs. 4.4 Ω·cm^2^) demonstrates a significant and reproducible improvement in corrosion resistance that correlates directly with the microstructural differences observed in SEM and TEM (Figure 3 and Figure 4). The 1400 nm coating exhibited an R value of 8.429 Ω, nearly double that of the 900 nm coating (4.369 Ω), suggesting its superior barrier performance. Bode phase angle plots (Figure 6b) show that the 1400 nm coating maintains a broader and higher peak phase angle, which indicates better capacitive behavior and a more stable passive coating. Similarly, the Bode impedance magnitude plots (Figure 6b) reveal higher |Z| values at low frequencies for the 1400 nm coating, which further supports its enhanced resistance to electrochemical attack. The fitted CPE-T and CPE-P values for both coatings were identical (1.4 × 10^−4^ and 0.8, respectively), suggesting comparable dielectric behavior but possible differences in surface uniformity or oxide density. The slightly better-defined arc in the Nyquist plot of the thicker coating may also reflect reduced surface roughness, consistent with SEM results. Both Nyquist plots exhibit a high-frequency intercept, a well-defined capacitive arc at intermediate frequencies (representing charge transfer), and a low-frequency tail indicative of diffusion-controlled behavior. This tail suggests that mass transport through corrosion products plays a role, particularly in thinner coatings. Overall, the EIS findings strongly corroborate the potentiodynamic polarization and SEM results, demonstrating that the 1400 nm coating provides superior electrochemical stability and corrosion resistance due to its denser, more continuous passive layer. The selection of a single time-constant equivalent circuit model was based on the characteristics of the EIS spectra themselves. As observed in the Bode phase plot (Figure 6b), the spectra for both coatings are dominated by a single, well-defined time constant, indicating that the responses from the coating and the double layer are not sufficiently separated in the frequency domain to be resolved as distinct features. This spectral behavior is consistent with EIS studies of dense metallic coatings where the coating does not exhibit distinct porosity-related capacitance [45].

**Figure 7:**
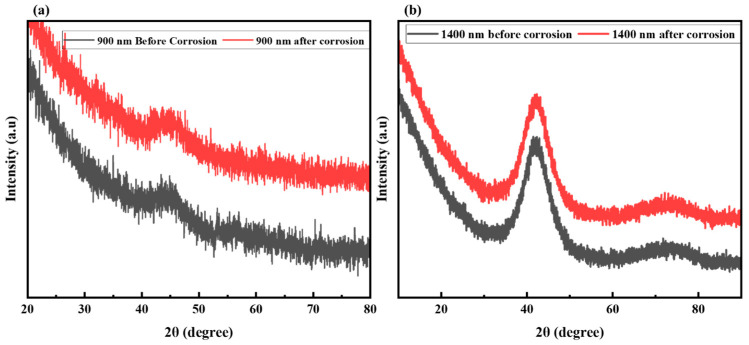
XRD patterns of Al_2_Cr_16_Fe_50_V_20_Ti_12_ HEA coatings before and after corrosion in 3.5 wt% NaCl: (**a**) 900 nm coating; (**b**) 1400 nm coating.

It is noteworthy to examine the XRD signatures of both coatings before and after exposure to a 3.5 wt% NaCl solution. Both the 900 nm and 1400 nm Al_2_Cr_16_Fe_50_V_20_Ti_12_ HEA coatings exhibit a broad amorphous hump centered around 2θ ≈ 40°, endorsing their predominantly non-crystalline structure as a result of the sputtering process, as shown in Figure 7. Post-corrosion patterns reveal subtle but distinct changes: a reduction in peak intensity and minor broadening are observed in both coatings, more noticeably in the 900 nm sample (Figure 7). No phase differentiation is observed in Figure 7 because both coatings are fully amorphous, as confirmed by SAED patterns insect in (Figure 4a,b). Amorphous materials produce only broad halos rather than sharp diffraction peaks. The absence of new peaks after corrosion indicates that no crystalline phases formed in detectable quantities and the amorphous structure remained stable. The subtle intensity reduction and broadening, particularly in the 900 nm coating, indicate surface disorder and corroborate the electrochemical findings. These changes suggest partial surface oxidation and increased structural disorder, likely due to localized corrosion effects [46]. In contrast, the 1400 nm coating retains its amorphous signature, with only minimal variations, indicating greater structural stability and resistance to chloride-induced degradation. This observation aligns with the electrochemical data and supports the conclusion that coating thickness plays a crucial role in improving corrosion resistance and maintaining microstructural integrity.

**Figure 8:**
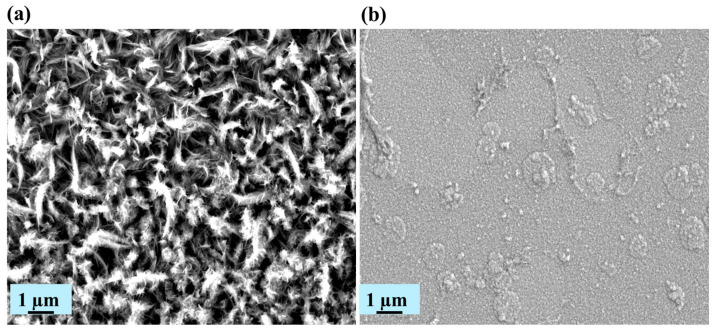
Surface SEM images of Al_2_Cr_16_Fe_50_V_20_Ti_12_ HEA coatings after corrosion in 3.5 wt% NaCl: (**a**) 900 nm, (**b**) 1400 nm.

Figure 8 presents the post-corrosion surface morphologies of the Al_2_Cr_16_Fe_50_V_20_Ti_12_ HEA coatings after immersion in 3.5 wt% NaCl solution. The 1400 nm coating (Figure 8a) retains a smooth and uniform surface with no visible signs of delamination, pitting, or surface cracking. This indicates excellent corrosion resistance and the preservation of surface integrity even under aggressive chloride exposure. In contrast, the 900 nm coating (Figure 8b) displays significant morphological degradation. The surface exhibits widespread roughening, pitting, and cracking, indicative of localized breakdown of the passive coating. These features suggest electrolyte penetration through defects or porous regions, leading to accelerated corrosion. The degradation is attributed to the limited ability of the thinner coating to develop a stable and compact passive layer, as supported by prior electrochemical and XRD findings (Figure 7). The negligible surface changes in the 1400 nm coating confirm its superior protective capability, which is likely due to increased coating thickness, higher density, reduced porosity, and a more robust passive oxide formation. These morphological observations strongly reinforce the earlier electrochemical results, underscoring the critical role of thickness in improving the corrosion performance of the HEA coating. The fitted CPE parameters provide valuable insight into the physical characteristics of the coating–electrolyte interface. Both coatings exhibited identical CPE-T (Q) values of 1.4 × 10^−4^ and CPE-P (*n*) values of 0.8. The CPE-P value of 0.8 indicates non-ideal capacitive behavior, which is typical for solid electrodes and can be attributed to surface roughness, heterogeneity, and variations in passive film composition or thickness [45,47]. According to Orazem et al. [45], CPE behavior in corrosion-resistant coatings arises from distributed time constants along the electrode surface, which correlate directly with surface topography and local variations in film resistivity. In our system, the identical CPE parameters suggest that the intrinsic dielectric properties and heterogeneity of the passive film formed on both coatings are similar, meaning the chemical composition and basic structure of the passive layer are comparable regardless of coating thickness. Therefore, the superior corrosion performance of the 1400 nm coating cannot be attributed to a fundamentally different passive film chemistry. Instead, the primary factor is its higher density and lower defect density, as confirmed by SEM (Figure 3) and TEM (Figure 4). The dense, continuous microstructure of the 1400 nm coating provides a larger effective area for stable passive layer formation and presents a more uniform surface with fewer weak points for chloride attack. This microstructural quality is directly reflected in the higher charge transfer resistance (Rct), while the CPE parameters capture the local heterogeneity that is similar for both coatings. As Gharbi et al. [47] demonstrated, the relationship between film thickness, dielectric properties, and CPE response is complex, and in our case, the identical CPE values reinforce that the improved performance of the 1400 nm coating is a morphological rather than compositional advantage.

**Figure 9:**
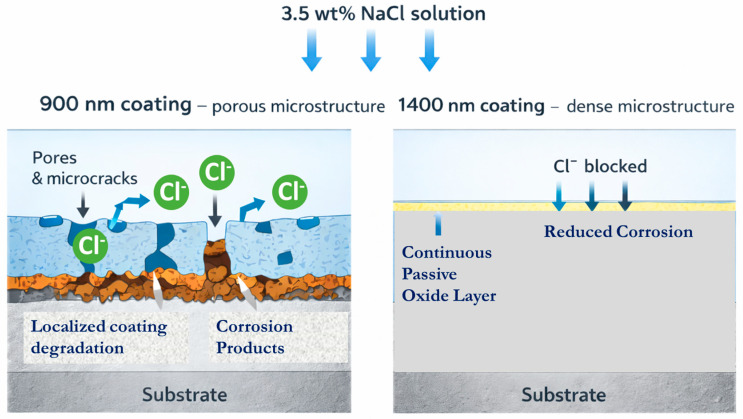
Corrosion mechanism in Al_2_Cr_16_Fe_50_V_20_Ti_12_ HEA coatings of different thicknesses exposed to NaCl.

Figure 9 schematically illustrates the proposed corrosion mechanism in the Al_2_Cr_16_Fe_50_V_20_Ti_12_ HEA coating of different thicknesses when exposed to a 3.5 wt% NaCl solution. In the 900 nm coating, localized corrosion is initiated by the incomplete coverage of the passive layer and the presence of surface irregularities, as confirmed by TEM and SEM. These discontinuities permit chloride ions (Cl^−^) to penetrate the surface and disrupt atomic bonding, leading to the formation of anodic and cathodic reaction sites. From a kinetic perspective, this process can be described by the Butler–Volmer equation:

(2) $$i=i_{0}\left[\exp\left(\frac{\alpha_{a}F\eta }{RT}\right)-\exp\left(\frac{-\alpha_{c}F\eta }{RT}\right)\right]$$

where *i* represents the net current density, *i*_0_ is the exchange current density, η is the overpotential, *α*_a_ and *α*_c_ are the transfer coefficients, F is the Faraday constant, R is the gas constant, and T is the temperature in Kelvin. A higher experimentally observed corrosion current density ($I_{corr}=44.6\;\mu A\cdot {cm}^{-2}$) for the 900 nm coating implies a higher $i_{0},$ possibly due to a larger electrochemically active area caused by surface defects.

In contrast, the 1400 nm coating develops a more uniform and continuous passive layer, supported by a denser microstructure with fewer interfacial gaps. This morphology restricts the ingress of Cl^−^ ions and minimizes anodic activity. The improved performance is further validated by EIS fitting, which shows a higher charge transfer resistance (R_ct_ = 8.429 Ω) and a better-defined capacitive arc in the Nyquist plot. These features are well-modeled by the EEC, where the interface is modeled as a parallel combination of a CPE and R_c_:

(3) $$Z_{CPE}=\frac{1}{{Q(j\omega )}^{n}}$$

Both coatings share similar CPE parameters (Q = 1.4 × 10^−4^, *n* = 0.8), but the larger R_ct_ in the 1400 nm coating suggests that the passive layer is denser or less defective, reducing the overall charge transfer rate.

Additionally, the corrosion barrier performance can be described using the classical capacitance–thickness relationship:

(4) $$C=\frac{\varepsilon \varepsilon_{0}A}{d}$$

where C represents the capacitance, d denotes the coating thickness, and ε is the dielectric constant. A lower capacitance, as implied by the EIS data, indirectly confirms a thicker or more compact passive layer in the 1400 nm coating. Finally, the diffusion-controlled tail in the Nyquist plots, more prominent in the 900 nm coating, can be linked to Fick’s second law:

(5) $$\frac{\partial C}{\partial t}=D\frac{\partial^{2}C}{{\partial x}^{2}}$$

This describes how chloride ions penetrate the porous structure of the 900 nm coating over time, contributing to its early-stage degradation. In contrast, the 1400 nm coating effectively impedes this transport due to its compact morphology. Figure 9 schematically illustrates the proposed corrosion mechanism in the Al_2_Cr_16_Fe_50_V_20_Ti_12_ HEA coating of different thicknesses when exposed to 3.5 wt% NaCl solution. The diagram correlates the microstructural observations from SEM and TEM (Figure 3 and Figure 4) with the electrochemical results from polarization and EIS testing (Figure 5 and Figure 6). In the 900 nm coating (left side of Figure 9), localized corrosion is initiated by the incomplete coverage of the passive layer and the presence of surface irregularities and porosity, as confirmed by cross-sectional SEM (Figure 3a) and TEM (Figure 4a insect). These discontinuities permit chloride ions (Cl^−^) to penetrate the surface and disrupt atomic bonding, creating anodic and cathodic reaction sites at the coating–substrate interface. The physical consequence of this defective structure is a larger electrochemically active surface area, which directly explains the higher experimentally observed corrosion current density (Icorr = 44.6 μA·cm^−2^) for the 900 nm coating. The Butler–Volmer equation relates this increased current density to a larger exchange current density (i_0_) resulting from the expanded active area.

In contrast, the 1400 nm coating (right side of Figure 9) develops a uniform and continuous passive layer supported by a dense, defect-free microstructure with complete coalescence (Figure 3b and Figure 4b insect). This morphology physically restricts the ingress of Cl^−^ ions, limits anodic activity to the outer surface, and prevents electrolyte penetration to the substrate. The improved barrier performance is quantitatively validated by EIS fitting, which shows a higher charge transfer resistance (Rct = 8.4 Ω·cm^2^) for the 1400 nm coating. In the equivalent circuit model, the interface is represented as a parallel combination of a constant phase element (CPE) and charge transfer resistance (Rct). The CPE captures the distributed time constants arising from surface heterogeneity [48], while Rct quantifies the kinetic barrier to charge transfer. Although both coatings share similar CPE parameters (Q = 1.4 × 10^−4^, *n* = 0.8), indicating comparable local dielectric properties of the passive film, the larger Rct in the 1400 nm coating confirms that its passive layer is physically denser and covers a more uniform surface, reducing the overall charge transfer rate. This interpretation is consistent with the capacitance–thickness relationship (C = εε_0_A/d), where the lower effective capacitance implied by EIS data for the 1400 nm coating is consistent with a more uniform film thickness distribution and fewer localized thin spots.

Finally, the diffusion-controlled tail observed in the Nyquist plots (Figure 6a), which is more prominent in the 900 nm coating, physically corresponds to chloride ion transport through the porous corrosion product layer and along coating defects. In the 900 nm coating, this transport follows Fickian diffusion kinetics through interconnected porosity, contributing to its early-stage degradation. In contrast, the 1400 nm coating effectively impedes this transport due to its compact morphology, eliminating the diffusion pathway to the substrate. This microstructure-based interpretation directly links the physical characteristics observed in electron microscopy with the quantitative parameters extracted from electrochemical measurements, demonstrating that coating thickness critically influences corrosion performance through its effect on density, defect population, and passive layer uniformity.

### 3.3. Mechanical Properties of Al_2_Cr_16_Fe_50_V_20_Ti_12_ Coatings

The 900 nm coating exhibits a porous and partially coalesced microstructure, as evidenced in Figure 3, resulting in significant heterogeneity in its deformation response during nanoindentation. The measured mechanical response is, therefore, dominated by void collapse and microstructural discontinuities rather than intrinsic coating properties. For this reason, quantitative mechanical analysis and FEM modeling were restricted to the dense and structurally continuous 1400 nm coating [49].

**Figure 10:**
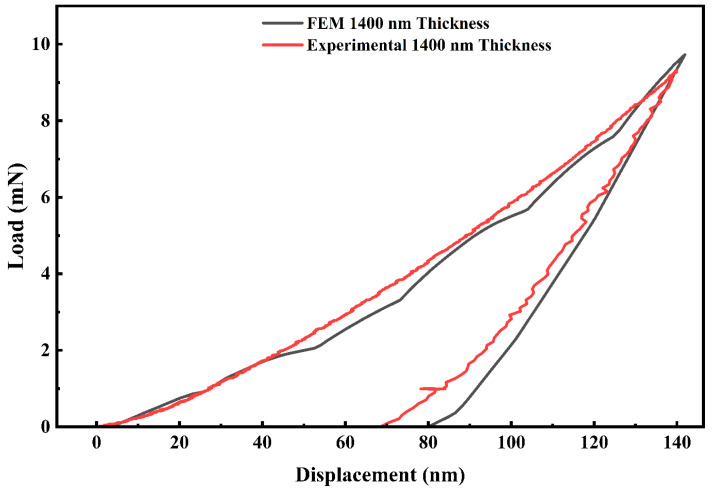
Load–displacement (P–h) curves from nanoindentation experiments and FEM simulations for Al_2_Cr_16_Fe_50_V_20_Ti_12_ HEA coatings of 1400 nm thicknesses.

The nanoindentation test revealed that the hardness of the 1400 nm-thick HEA film was 10.9 ± 0.5 GPA, consistent with earlier studies [1]. As displayed in Figure 10, the numerical outcomes exhibit strong agreement with the experimental curve across the full range of indentation depths, suggesting the suitability of the adopted model geometry, boundary conditions, and material property inputs for simulating nanoindentation of the HEA coating. The minor deviations observed in the initial portion of the unloading segments, primarily in the low-force regime, are likely due to modeling simplifications, including the assumption of an elastic–perfectly plastic material response and the idealized geometries of the tip of the indenter and the coating surface. Despite these idealizations, the overall behavior of the simulated curves aligns well with experimental observations. Prior work (e.g., [50]) has shown that critical indentation response characteristics, such as penetration depth and curve shape, are more prone to differences in elastic features than to plastic ones. Moreover, the use of yield stress as a primary input parameter in similar thin-coating studies has been widely accepted and validated [51]. Therefore, the current model can be considered both accurate and robust for extracting mechanical response characteristics such as yield strength and stress–strain distributions in the focus area.

Based on the best-fitting FEM curves, the 1400 nm coating exhibited a yield strength of 7 to 8 GPa, as outlined in Table 4.

**Figure 11:**
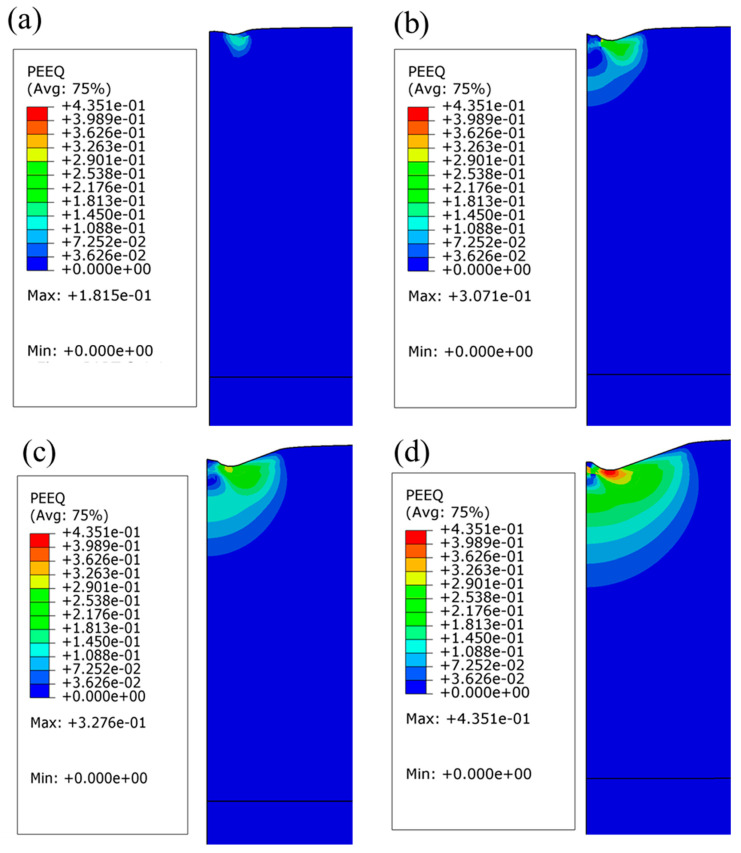
Equivalent plastic strain distribution in the 1400 nm-thick Al_2_Cr_16_Fe_50_V_20_Ti_12_ HEA coating at indentation depths of (**a**) 30 nm; (**b**) 60 nm; (**c**) 90 nm; and (**d**) 140 nm.

**Figure 12:**
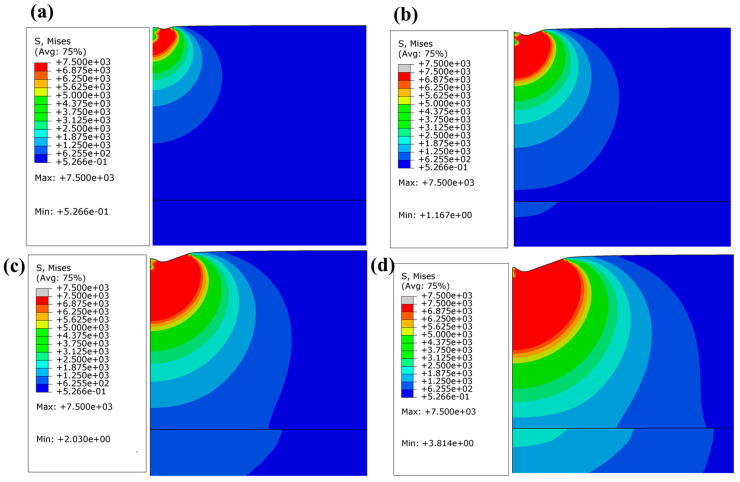
Von Mises stress distribution in the 1400 nm-thick Al_2_Cr_16_Fe_50_V_20_Ti_12_ HEA coating at indentation depths of (**a**) 30 nm; (**b**) 60 nm; (**c**) 90 nm; and (**d**) 140 nm.

Figure 11 and Figure 12 provide complementary insights into the mechanical response of the 1400 nm Al_2_Cr_16_Fe_50_V_20_Ti_12_ HEA coating under nanoindentation via equivalent plastic strain (PEEQ) and von Mises stress (σ_max_) distributions, respectively.

The PEEQ contours at indentation depths of 30, 60, 90, and 140 nm (Figure 11a–d) show the plastic zone expanding progressively and symmetrically into a hemispherical shape. At shallow depths, deformation is highly localized beneath the indenter, but at greater depths, the zone penetrates deeper and broadens laterally. Even at 140 nm, the plastic strain remains confined within the coating, indicating negligible substrate influence. Peak PEEQ increases from ≈0.18 to ≈0.44, confirming the ability of the 1400 nm coating to accommodate significant localized deformation and indicating high ductility and toughness. This unique deformation tolerance aligns with observations in other HEAs, where the synergy of dislocation activity, stacking-fault formation, nanobridge-mediated crack arrest, and complex phase interactions yields both high strength and ductility [52].

Figure 12a–d present the von Mises stress contours across the same indentation depths. At shallow penetration, stresses are sharply concentrated near the surface, hinting at the onset of localized yielding. With increased depth, the stress field becomes broader and more uniform, assuming a hemispherical distribution while maintaining σ_max_ ≈ 7.5 GPa. Importantly, stress remains fully contained within the film thickness, which suggests robust stress redistribution, with no signs of brittle failure or delamination. Such mechanical resilience is typical of HEA coatings under extreme loading or irradiation; their compositional complexity and defect-tolerant structures facilitate stress accommodation without catastrophic fracture (e.g., in radiation-tolerant HEAs and deposition-based HEA films) [53]. The strain and stress distributions collectively provide an understanding of the mechanical behavior of the coating, with the strain contours illustrating the evolution of plastic deformation and the stress contours revealing how the coating manages the applied loads. This dual representation enhances interpretation of experimental nanoindentation data and features the intrinsic mechanical resilience of the Al_2_Cr_16_Fe_50_V_20_Ti_12_ HEA coating, supporting its suitability for applications demanding both ductility and stability, such as radiation-tolerant barrier coatings in nuclear environments [52,53,54].

## 4. Conclusions and Future Recommendations

This study explores the electrochemical behavior of novel Al_2_Cr_16_Fe_50_V_20_Ti_12_ HEA coatings with thicknesses of 900 nm and 1400 nm deposited via magnetron sputtering. Based on the comprehensive corrosion analysis, it is concluded that the 1400 nm-thick Al_2_Cr_16_Fe_50_V_20_Ti_12_ HEA coating provides exceptional corrosion resistance in 3.5 wt% NaCl solution, markedly outperforming its 900 nm counterpart. The superior performance is directly attributed to the critical role of coating thickness in achieving a dense, continuous, and fully amorphous microstructure. This optimized structure acts as an effective barrier, minimizing defects and facilitating the formation of a stable passive layer, as evidenced by a tenfold reduction in corrosion current density, a doubled charge-transfer resistance, and the preservation of surface and structural integrity post-exposure. This outstanding corrosion resistance is complemented by robust mechanical properties, including a hardness of 10.9 ± 0.5 GPa and a high yield strength of 7–8 GPa, as determined by nanoindentation and simulation. The combined resistance to both corrosive degradation and mechanical deformation underscores the significant potential of these coatings for applications in harsh environments, particularly in lead–bismuth eutectic (LBE)-cooled nuclear systems. Future research should focus on optimizing deposition parameters to further enhance these properties, evaluating performance under irradiation, and exploring corrosion behavior in a broader range of simulated service environments.

## Figures and Tables

**Table 1:** Processing parameters for synthesis of coatings.

S. No	Coating Thickness (nm)	Power (Watt)	Time (h)	Argon Pressure (sccm)	Working Pressure (Pa)
1	900	50	2	80	0.3
2	1400	50	3	80	0.3

**Table 3:** Composition of Al_2_Cr_16_Fe_50_V_20_Ti_12_ HEA target and coating as determined by EDS.

Element	Al	Ti	Cr	Fe	V
Target (at.%)	3.73	13.76	13.82	48.57	20.12
1400 nm	2.66	11.14	16.18	50.56	19.19
900 nm	1.33	11.19	16.17	50.92	18.31

**Table 4:** Mechanical property ranges of HEA coating.

Coating Thickness	Material	Property Ranges Observed in Analysis
1400 nm	Al_2_Cr_16_Fe_50_V_20_Ti_12_	Young’s modulus: 150–170 GPa Initial yield stress: 6–8 GPa Poisson’s ratio: 0.32

## Data Availability

The original contributions presented in this study are included in the article. Further inquiries can be directed to the corresponding authors.
